# Returning home: forced conscription, reintegration, and mental health status of former abductees of the Lord's Resistance Army in northern Uganda

**DOI:** 10.1186/1471-244X-9-23

**Published:** 2009-05-16

**Authors:** Phuong N Pham, Patrick Vinck, Eric Stover

**Affiliations:** 1 Human Rights Center, University of California, Berkeley, Berkeley, CA, USA; 2 Payson Center for International Development, Tulane University, New Orleans, LA, USA; 3 Human Rights Center, School of Public Health, and School of Law, University of California, Berkeley, Berkeley, CA, USA

## Abstract

**Background:**

Since the late 1980s, the Lord's Resistance Army (LRA), a spiritualist rebel group in northern Uganda, has killed and mutilated thousands of civilians and abducted an estimated 52,000 to 75,000 people to serve as soldiers, porters, and sex slaves for its commanders. This study examines the types of violence to which former abductees have been exposed and the extent to which these acts have affected their psychological well-being.

**Methods:**

This is a cross-sectional study of 2,875 individuals selected through a multi-stage stratified cluster sampling design conducted in 8 districts of northern Uganda. Multivariate logistic regressions were performed with symptoms for Post-traumatic Stress Disorder (PTSD) and depression as the main outcome measures.

**Results:**

One-third of the respondents (33%) self-reported having experienced abduction (49% among the Acholi, the largest tribal group in northern Uganda). Over half (56%) of all the respondents and over two-thirds of those who experienced abduction met the criteria for symptoms of post-traumatic stress disorder (PTSD). Multivariate analysis shows that several factors increased the risk of former LRA abductees developing symptoms of PTSD. These factors included gender (females were more susceptible than males), being a member of the Acholi ethnic group, participating in or witnessing a cumulative number of traumatic events, and encountering difficulties re-integrating into communities after abduction. Factors associated with increased risk of meeting criteria for symptoms of depression included older age of males at the time of abduction, lower score on social relationship scale, high incidence of general traumatic event exposure, high incidence of forced acts of violence, and problems reintegrating into communities after abduction.

**Conclusion:**

Abduction and forced conscription of civilians has affected the psychological well-being of a significant number of northern Ugandans. The sources of psychological trauma are multiple, ranging from witnessing to being forced to commit violent acts, and compounded by prolonged exposure to violence, often for months or years. Community-based mental health care services and reintegration programs are needed to facilitate the reintegration of former abductees back into their communities.

## Background

Twenty-one years of war, destruction, and the displacement of over 1.5 million people have turned northern Uganda into a humanitarian disaster. One of the principal belligerents in the conflict has been the Lord's Resistance Army (LRA), a spiritualist rebel group that has killed and mutilated thousands of civilians and abducted an estimated 52,000 to 75,000 children and adults to serve as soldiers, porters, and sex slaves for its commanders [1]. In response, the International Criminal Court (ICC) issued warrants of arrest, on 13 October, 2005, against LRA leader Joseph Kony and four of his top commanders for crimes against humanity and war crimes, including the forced conscription of children [2]. Within weeks, the LRA withdrew its forces to the southern Sudan and then crossed the Nile, assembling in Garamba National Park in the Democratic Republic of Congo. In the summer of 2006, peace talks between the Government of Uganda and the LRA commenced in Juba, Sudan but collapsed eighteen months later when Kony refused to sign a final peace agreement. By February 2009, hundreds of thousands of Ugandans remained in displacement camps throughout the North and, in eastern Congo, the LRA rebels and joint Ugandan-Congolese troops were engaged in armed skirmishes.

While abduction of children and youth into regular and rebel armies has been a common feature of recent armed conflicts (Sri Lanka, Nepal, Angola, Mozambique, Sierra Leone, Liberia, Uganda, Burma), little is known about the process of reintegrating former abductees back into their communities [3]. Until early 2007, community and international humanitarian organizations in northern Uganda had operated 12 reception centers for LRA abductees who were either captured in battle or managed to flee their captors [1,4-6]. Upon arrival, former abductees were given a medical exam and treated for diseases and other ailments. Those suffering from war wounds were sent to hospitals in Gulu and Kampala. Most returnees stayed at the centers for two to six weeks were the participated in a range of activities, including counseling, music and dance, sports, and vocational training. During that time, staff members attempt to trace the whereabouts of their parents and relatives and, if successful, the former abductees would be reunited with their parents or other relatives.

To understand how abduction and the process of reintegration had affected former LRA combatants we analyzed a cross-sectional survey that was conducted in eight districts of northern Uganda between March and June 2007.

## Methods

### Survey Sites and Sample Selection

**Figure 1:**
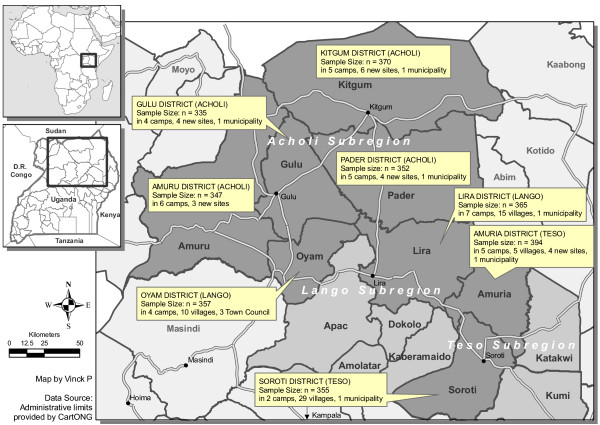
**Eight sampled districts in northern Uganda**.

Study participants were Ugandan adults (18 years of age or older) randomly selected using a multi-stage sampling strategy. The Committee for Protection of Human Subjects at Tulane University and University of California, Berkeley, and northern Uganda local government officials approved the research protocol. No incentive was provided to the survey participants. The districts were selected to represent a variety of ethnic groups (Acholi, Iteso, and Langi) and exposure to the armed conflict (Figure 1). The resulting minimum sample size–320 individuals for each district–was determined using the difference in proportion formula. The sample size was adjusted for stratification and design effect due to cluster sampling and missing responses. The assumed level of precision was 10% with 80% power. Within each district, camps for internally displaced people were randomly selected using a sampling technique proportionate to population size. In some cases, residents of the camps had recently moved to new settlement sites closer to their original villages. In order to capture this population, we randomly selected one new settlement site for each of the selected camps where population movement had taken place, based on the database provided by the World Food Programme (WFP) and the United Nations High Commission for Refugees (UNHCR). In areas where the population was never displaced or had returned to live in villages, sub-counties were first sampled using a sampling technique proportionate to population size, then parishes and then villages. In the camps and villages, interviewers were assigned to zones of approximately equal size where they selected every other household in a randomly chosen direction. A household was defined as a group of people normally sleeping under the same roof and eating together. In each household, interviewers randomly selected one adult respondent, of the same gender as the interviewer, from a list of all eligible adults. Three attempts were made to contact a household or individual.

Three teams of eight to 16 local university students or graduates with experience in survey work participated in a week-long training to familiarize themselves with the standardized pre-coded open-ended questionnaire, interview techniques, and selection process for respondents. The teams were composed of equal numbers of men and women, represented the ethnic group of the area under study and were fluent in the local language. The training included a pilot survey in non-sampled sites. Data were collected using Personal Digital Assistants (PDA) with integrated Global Positioning System (GPS). The interviewers attended an additional three day of training on the use of the PDA.

Interviewers approached a total of 4,455 households and 2,875 individuals were ultimately interviewed. One third (35%) of the households was replaced by the next selected household either because the household was empty and no one could be contacted after three attempts (64%), because no one was eligible in the household (no one 18 years of age or older (24%), or because they refused to participate (12%). Within the selected households, one individual was randomly selected. If that person could not participate, another respondent within the same household was selected. In total, 20 of the selected individuals had to be replaced either because they were absent or could not be contacted after three attempts (78%) or because they refused to participate (22%). Two interviews were conducted mistakenly with individuals aged below 18 years old and were not included in the analysis. Eleven interviews were not completed but the completed responses are nevertheless included in the analysis. The final sample size for the eight districts was 2,875 individuals in 38 camps, 21 new sites, 59 villages, and nine municipalities or town councils. The sample was distributed as follow (by district): Amuru: 347; Gulu: 335; Kitgum: 370; Pader: 352; Lira: 365; Oyam: 357; Amuria: 394; Soroti: 355.

### Research Instruments and Data Entry

The survey instrument covered 15 topics and was translated into the three local languages. Back-translation and consultation with local experts was used to ensure the quality of the translation. The questionnaire was field tested and validated in non-participating sites and mock interviews were organized during the training of the interviewers.

Response options were provided to the interviewer but not read to the participant unless otherwise indicated. An "other" category was available to record responses when necessary or when the interviewers were unsure of the appropriate response option; it was recoded during analysis. Each evening the data were synchronized with a central MySQL database and records were manually checked for errors. One-on-one interviews were conducted anonymously in a confidential setting. Due to the sensitivity of some of the questions, the interviewers were assigned to same-sex respondents. Oral rather than written consent was obtained due to the high illiteracy rate. The consent form stressed confidentiality and respondents' names were never recorded.

### Measurements and Data Analysis

Digital data from the interviews were imported and analyzed using Statistical Package for Social Science (SPSS) version 15.0. No weights were applied for the present study. Scores for the 17-item PTSD Checklist-Civilian Version (PCL-C), a measure of post-traumatic stress disorder, and 15-item depression section of the Johns Hopkins Depression Symptom Checklist (JHD) were computed to assess symptoms of PTSD (cutoff score of 44) and depression (cutoff score of 42), respectively. The PCL-C, which has been correlated with the Clinician-Administered PTSD Scale (CAPS), uses simple language that eases the process of translation and administration by nonclinicians to a population with low levels of education, and has been shown to have good internal reliability and high convergent validity in a wide variety of studies [7]. In addition, the PCL-C and JHD had good reliability when used by the investigators for this and prior study in Uganda [8] and other countries including Rwanda and Democratic Republic of Congo [9-11]. The estimated Cronbach α (a measure of internal reliability) for the three PCL-C symptom clusters in this study were as follows: re-experiencing, α = .875; avoidance, α = .860; and hyperarousal, α = .850. The estimated Cronbach α for the Depression Symptom checklist was α = .927.

Building on previous research in northern Uganda [8], we developed a list of 33 items to measure exposure to violence and experience of abduction. The list does not represent all possible traumatic events but rather focuses on commonly reported events. Building on measurements of exposure to violence [12], four summative scales were built to measure exposure to different categories of violent events: direct victim of violence (e.g., being beaten); witness to violence; secondary exposure (e.g., loss of a family member); and forced use of violence (e.g., being forced to loot or beat someone).

We developed a measure of social relationship based on three questions asking respondents to rank their relationship with their family, friends and neighbors, and community in general on a five-point Likert scale. Principal component analysis was used to analyze the three items and resulted in one factor explaining 74.1% of the original variance. A summative scale based on the original items was therefore used as a measure of social relationship (Cronbach α = .820).

We performed two separate multivariate logistic regressions to examine factors associated with psychological disorders (symptoms of PTSD and depression) among respondents who reported experiencing abduction. The predictors of greatest interest were exposure to violence (summative scale by patterns of exposure), length of abduction, going through a reception center, and social relationship. Logistic regressions analysis allows one to compute an odds ratio, an estimate of relative risk, and is easier to interpret in exploring the complex relationships presented in this paper. We performed both forward- and backward-stepwise hierarchal regressions. Only the statistically significant predictors were included in the final models.

## Results

### General Characteristics of Abductees

Of 2,867 respondents with complete information on experience of abduction, 946 (33%) reported they had been abducted at one time or another during the course of the 20-year conflict in northern Uganda. Among them, 46% stated that they had been abducted on two or more occasions. In the Acholi sub-region, almost half of the respondents (49%) stated that they had been abducted compared to one-fifth (22%) in the Lango subregion and one-tenth (11%) in the Teso subregion. This pattern is reflected in the proportion of former abductees by ethnic group since ethnic distribution roughly follows administrative limits.

As shown in Table 1 women accounted for 410 (43%) of the 946 respondents who reported experiencing abduction. Males were more likely to have reported abduction (OR_undj _= 1.44, 95% C.I. = 1.23, 1.68) compared to females. The mean age of respondents who reported abduction was 35.3 at the time of the survey (S.D. 13.51) similar to the mean age of those who did not report abduction (35.2). At the time of their first abduction, respondents who were held in captivity averaged 25.8 years old (S.D. 13.57). A majority of those who were held in captivity were in a committed relationship, either married (71.8%) or in a partnership (3.6%). About a quarter (24.8%) of the abducted had no education and 40% had some but incomplete primary education. Formerly abducted respondents further self-identified as Catholic (70.2%) more frequently than non-abducted respondents (57.8%).

**Table 1:** Socio-Demographic Profile of Respondents by Abduction Status

		All	Not Abducted	Abducted
n		2,867	1,921	946
		(100%)	(67%)	(33%)

Gender (women)		1,417	1,007	410
		(49.5%)	(52.4%)	(43.4%)

Mean Age		35.4	35.42	35.26
(S.D.)		(14.35)	(14.75	(13.51)

Mean Age at Abduction		-	-	25.84
(S.D.)		-	-	(13.57)

Mean Income, Ugandan Shillings (S.D.)		33,426	35,804	28,701
		(166,885)	(199,492	(60,874)

**Ethnicity**	Acholi	1,385	706	679
		(48.3%)	(36.7%)	(71.8%)
	
	Langi	712	633	79
		(24.8%)	(32.9%)	(8.4%)
	
	Teso	724	540	184
		(25.2%)	(28.1%)	(19.5%)
	
	Other	47	43	4
		(1.6%)	(2.2%)	(0.4%)

**Marital Status**	Single	373	265	108
		(13.0%)	(13.8%)	(11.4%)
	
	Married	2,070	1,391	679
		(72.2%)	(72.4%)	(71.8%)
	
	Partner	77	43	34
		(2.7%)	(2.2%)	(3.6%)
	
	Divorced	130	78	52
		(4.5%)	(4.1%)	(5.5%)
	
	Widowed	217	144	73
		(7.6%)	(7.5%)	(7.7%)

**Religion**	Catholic	1,771	1,108	663
		(61.9%)	(57.8%)	(70.2%)
	
	Protestant	802	580	222
		(28.0%)	(30.2%)	(23.5%)
	
	Born Again	227	177	50
		(7.9%)	(9.2%)	(5.3%)
	
	Other	63	53	10
		(2.2%)	(2.8%)	(1.1%)

**Education**	No schooling	698	473	225
		(24.3%)	(24.6%)	(23.8%)
	
	Some primary	1,073	691	382
		(37.4%)	(36.0%)	(40.4%)
	
	Completed primary	459	314	145
		(16.0%)	(16.3%)	(15.3%)
	
	Higher than primary	638	444	194
		(22.2%)	(23.1%)	(20.5%)

Of those who reported abduction, 426 (45%) were held for less than a day, 199 (21%) were held captive for one to seven days, and 122 (13%) were held for between one week and one month. One hundred and two respondents (11%) were held for about one to six months and 97 (10%) for more than six months.

### Exposure to Traumatic Events and War Crimes

Exposures to four categories of violent traumatic events were assessed: direct victim (e.g., being beaten), witness to violence, secondary exposure (e.g., loss of a family member), and forced to use violence (e.g., being forced to loot or beat someone). While exposure to violence was widespread among respondents, former LRA abductees reported higher level of exposure in all four categories (see Table 2). The average cumulative number of reported events within each category was also higher among former LRA abductees (p-value < .001).

**Table 2:** Exposure to Traumatic Events and War Crimes

	All	Not Abducted	Abducted
**Direct Violent Exposure**			

Was displaced	2,450	1,546	904
	(85.4%)	(80.4%)	(95.6%)

Lost income due to conflict	2,441	1,562	879
	(85.1%)	(81.3%)	(92.9%)

House destroyed	2,412	1,505	907
	(84.1%)	(78.3%)	(95.9%)

Lost productive assets	2,387	1,490	897
	(83.2%)	(77.5%)	(94.8%)

Was beaten by the LRA	587	57	540
	(20.8%)	(3.0%)	(57.1%)

Was maimed by the LRA	173	23	150
	(6.0%)	(1.2%)	(15.9%)

Was sexually violated	61	22	39
	(2.1%)	(1.1%)	(4.1%)

Problem with UPDF	645	369	276
	(22.5%)	(19.2%)	(29.2%)

Forced by LRA to walk long distances	833	89	744
	(29.0%)	(4.6%)	(78.6%)

Forced by LRA to carry loads	727	26	701
	(25.30%)	(1.4%)	(74.1%)

Mean Cumulative General Exposure (S.D.)*	4.94	4.16	6.61
	(2.00)	(1.79)	(1.28)

**Exposure as Witness**			

Witnessed an attack by LRA	1,988	1,114	874
	(69.3%)	(58.0%)	(92.4%)

Witnessed LRA/UPDF fight	1,259	653	606
	(43.9%)	(34.0%)	(64.1%)

Witnessed an abduction	1,812	935	877
	(63.2%)	(48.6%)	(92.7%)

Witnessed s.o. beaten by LRA	1,531	718	813
	(53.4%)	(37.4%)	(85.9%)

Witnessed s.o. beaten by UPDF	1,187	662	525
	(41.4%)	(34.4%)	(55.5%)

Witnessed s.o. killed by the LRA	931	406	525
	(32.5%)	(21.1%)	(55.5%)

Witnessed HH member killed by LRA	709	307	402
	(24.7%)	(16.0%)	(42.5%)

Witnessed s.o. sexually violated by LRA	249	84	165
	(8.7%)	(4.4%)	(17.4%)

Witnessed s.o. sexually violated by other	243	111	132
	(8.5%)	(5.8%)	(14.0%)

Mean Cumulative Witness Exposure (S.D.)*	3	2.47	5.19
	(2.57)	(2.43)	(1.86)

**Secondary Exposure**			

At least 1 family member killed	2,115	1,272	843
	(73.7%)	(66.2%)	(89.1%)

Spouse/Partner killed	88	53	35
	(3.1%)	(2.8%)	(3.7%)

Mother killed	141	73	68
	(4.9%)	(3.8%)	(7.2%)

Father killed	304	159	145
	(10.6%)	(8.3%)	(15.3%)

Brother killed	870	466	404
	(30.3%)	(24.2%)	(42.7%)

Sister killed	257	132	125
	(9.0%)	(6.9%)	(13.2%)

Son killed	226	132	94
	(7.9%)	(6.9%)	(9.9%)

Daughter killed	72	41	31
	(2.5%)	(2.1%)	(3.3%)

Grandmother killed	74	48	26
	(2.6%)	(2.5%)	(2.7%)

Grandfather killed	117	83	34
	(4.1%)	(4.3%)	(3.6%)

Family member was abducted	1,106	582	524
	(38.6%)	(30.3%)	(55.6%)

*Mean Cumulative Secondary Exposure (S.D.)*	1.15	0.93	1.61
	(1.10)	(1.04)	(1.10)

**Forced Acts of Violence During Captivity by the LRA**			

Forced to loot	NA	NA	353
			(37.2%)

Forced to beat or injure someone	NA	NA	167
			(17.6%)

Forced to kill someone	NA	NA	76
			(7.6%)

*Mean Cumulative Forced Acts of Violence (S.D.)*	NA	NA	0.62
			(0.92)

*p-value < .001 for difference in mean cumulative exposure between abducted and non-abducted people

### Returning home

Among those who were abducted less than one day, 67% reported they were released by the LRA compared to 32% of those held captive one to seven days and 18 among those held captive between eight days and one month (see Table 3). Conversely, 78% of those who were held for six months or more escaped compared to 57% among those captive one to seven days and 26% among those abducted less than one day.

**Table 3:** Experiences Returning Home among Former Lord's Resistance Army Abductees

	Abducted				
	
	< 1 day	1–7 days	8 days – < 1 month	1 – < 6 months	> 6 months
**Means of Return**					

Released by LRA	282	63	22	11	6
	(67.0%)	(32.0%)	(18.3%)	(11.2%)	(6.3%)

Escaped	111	112	80	79	74
	(26.4%)	(56.9%)	(66.7%)	(80.6%)	(77.9%)

Rescued by UPDF	26	22	17	8	14
	(6.2%)	(11.2%)	(14.2%)	(8.2%)	(14.7%)

Other	2	0	1	0	1
	(0.5%)	(0.0%)	(0.8%)	(0.0%)	(1.1%)

**Went through Reception center**	8	22	21	28	47
	(1.9%)	(11.0%)	(17.2%)	(27.7%)	(48.5%)

Helped return in community	2	20	20	23	46
	(25.0%)	(90.9%)	(95.2%)	(82.1%)	(97.9%)

Was visited by center's staff after return	1	10	14	13	23
	(12.5%)	(45.5%)	(66.7%)	(46.4%)	(48.9%)

**Had Problem Returning Home**	104	74	67	60	66
	(24.4%)	(37.0%)	(54.9%)	(59.4%)	(68.0%)

**Type of Problems**					

Stigmatization	4	34	7	10	12
	(4.0%)	(4.20%)	(11.30%)	(16.7%)	(18.2%)

"Mentally do not feel well"	10	8	8	5	7
	(9.9%)	(11.3%)	(12.9%)	(8.3%)	(10.6%)

Problem with adjusting to life outside the bush	8	3	9	10	11
	(7.9%)	(4.2%)	(14.5%)	(16.7%)	(16.7%)

Difficulty with school, work	9	7	4	7	13
	(8.9%)	(9.9%)	(6.5%)	(11.7%)	(19.7%)

Relation problems, family	9	9	9	6	6
	(8.9%)	(12.7%)	(14.5%)	(10.0%)	(9.1%)

Relations problems, friends/neighbors	6	3	8	4	3
	(5.9%)	(4.2%)	(12.9%)	(6.7%)	(4.5%)

Health, injury	18	26	8	9	7
	(17.8%)	(36.6%)	(12.9%)	(15.0%)	(10.6%)

Loss of property/goods	17	8	6	5	2
	(16.8%)	(11.3%)	(9.7%)	(8.3%)	(3.0%)

Other	20	4	3	4	5
	(19.8%)	(5.6%)	(4.8%)	(6.7%)	(7.6%)

Thirteen percent of the respondents said they had spent time in a reception center (see Table 3). One half (49%) of those abducted for six months or more reported to a reception center, compared to 2% of those abducted for a day or less. Overall, men were 1.76 times more likely to report that they had gone through a reception center than women (OR = 1.77, 95% C.I. = 1.18, 2.63, p-value = .005). However, the proportion of women going through reception centers was higher than that of men, at 52% compared to 47% (*χ*^2 ^= 7.96, df = 1, p-value = 0.005). Among those who went through a reception center, four out of five (88%) reported that the reception center helped them return to their communities, and almost half reported that they received follow-up visits from reception center staff.

Thirty-nine percent of former LRA abductees reported problems upon returning to their home communities. In addition, former LRA abductees who spent six or more months with the rebels (68%) reported more problems after returning home than those who stayed less time. While physical and material concerns were frequently mentioned ("health" and "injury": 18.9%; "loss of property" and "goods": 10.6%), most of those who returned reported mental and social problems ("mentally do not feel well": 10.6%; "problems adjusting to life outside the bush": 16.7%; "relationship problems with family": 10.8%).

### Symptoms of PTSD and Depression Among Former Abductees

Among respondents with a complete response to all items on the PCL-C and the Johns Hopkins Symptom Checklist who reported being abducted, 67% met the criteria for symptoms of PTSD and 40% met the criteria for symptom depression, compared to 51% and 25.9% respectively among those who were not abducted (see Table 4). Compared to non-abductees, those abducted were twice as likely to meet the criteria for symptoms of PTSD (OR_undj _= 2.12, 95% C.I. = 1.81, 2.51) and symptoms of depression (OR_undj _= 2.07, 95% C.I. = 1.75, 2.45). Respondents abducted for six months or more frequently met the criteria for symptoms of PTSD (80%) and symptoms of depression (47%) than those abducted for shorter periods.

**Table 4:** Psychosocial Well-being Among Former Lord's Resistance Army Abductees

	Not Abducted	Abducted	Abducted				
			
			< 1 day	1–7 days	8 days – < 1 month	1 – < 6 months	>6 months
**Symptoms of Depression**							

Total	481	373	160	77	55	36	45
	(25.9%)	(40.4%)	(38.6%)	(39.5%)	(47.0%)	(35.3%)	(47.4%)

Men	125	146	38	34	29	17	28
	(14.0%)	(27.8%)	(20.0%)	(28.8%)	(35.8%)	(24.6%)	(41.2%)

Women	355	227	122	43	26	19	17
	(36.8%)	(57.3%)	(54.7%)	(55.8%)	(72.2%)	(57.6%)	(63.0%)

**Total Symptoms for PTSD**							

Total	938	613	259	126	83	70	75
	(50.6%)	(67.1%)	(62.7%)	(65.3%)	(72.8%)	(70.7%)	(79.8%)

Men	306	274	73	57	51	42	51
	(34.6%)	(52.6%)	(38.8%)	(48.7%)	(63.8%)	(61.8%)	(75.0%)

Women	630	338	185	69	32	28	24
	(65.2%)	(86.4%)	(82.6%)	(90.8%)	(94.1%)	(90.3%)	(92.3%)

Cluster: Reexperiencing	1,351	763	334	161	108	79	81
	(71.3%)	(81.5%)	(79.0%)	(82.6%)	(90.0%)	(78.2%)	(83.5%)

Cluster: Avoidance/numbing	861	563	233	118	77	62	73
	(45.5%)	(60.4%)	(55.3%)	(59.6%)	(65.3%)	(61.4%)	(77.7%)

Cluster: Hyperarousal	1,079	676	299	141	87	75	74
	(56.8%)	(72.5%)	(71.0%)	(71.6%)	(73.7%)	(74.3%)	(77.1%)

**Relationship with family and community, Mean (S.D.)**	12.01	12.26	12.4	12.26	12.04	12.03	12.08
	(2.01)	(1.88)	(1.78)	(1.74)	(1.94)	(2.09)	(2.19)

After statistically controlling for the effect of other variables by employing multivariate logistic regression, reporting symptoms of PTSD was associated with gender, ethnicity, problems returning home, cumulative exposure as a witness, and cumulative exposure to forced acts of violence (see Table 5). Females were almost nine times more likely to report symptoms of PTSD (OR_adj _= 8.84, 95% C.I. = 6.07, 12.88). Among abductees, Acholi were three times more likely to meet symptom criteria for PTSD than Iteso (OR_adj _= 3.05, 95% C.I. = 1.59, 5.87). Langi were twice as likely to meet symptom criteria for PTSD than Iteso (OR_adj _= 2.15, 95% C.I. = 1.05, 4.41). There was no significant difference between Acholi and Langi respondents. Cumulative number of traumatic events witnessed (OR_adj _= 1.21, 95% C.I. = 1.10, 1.32) and cumulative number of forced acts of violence (OR_adj _= 1.43, 95% C.I. = 1.18, 1.74) among abductees were associated with meeting criteria for symptoms of PTSD. Finally, former abductees who reported difficulties coming home to their community after abduction were nearly three times more likely to meet criteria for symptoms of PTSD at the time of the survey (OR_adj _= 2.97, 95% C.I. = 2.09, 4.24).

**Table 5:** Statistically Significant Associations of Socio-demographic and Exposure Characteristics with Symptoms of PTSD and Depression

	**Symptoms of PTSD**		**Symptoms of Depression**	
Independent Variables	Adjusted OR	p-value	Adjusted OR	p-value
	(95% CI)		(95% CI)	

Gender				
Female/Male	8.84 (6.07, 12.88)	< 0.001	2.11 (1.22, 3.63)	0.007

Ethnicity				
Acholi/Iteso	3.05 (1.59, 5.87)	0.001	NS	NS
Langi/Iteso	2.15 (1.05, 4.41)	0.038	NS	NS
Acholi/Langi	1.42 (0.94, 2.16)	0.098	NS	NS
Acholi/Others	0.55 (0.65, 4.60)	0.578	NS	NS

Relationship with Family and Community	NS	NS	0.90 (0.83, 0.97)	0.007

Reported Problem Coming Home	2.97 (2.09, 4.24)	< 0.001	2.24 (1.63, 3.08)	< 0.001

Cumulative General Traumatic Exposure	NS	NS	1.18 (1.03, 1.33)	< 0.001
Cumulative Exposure as Witness	1.21 (1.10, 1.32)	< 0.001	NS	NS
Cumulative Forced Act of Violence	1.43 (1.18, 1.74)	< 0.001	2.24 (1.63, 3.08)	<0.001

Male* Age of Abduction	NS	NS	1.03 (1.01, 1.05)	0.001
Female*Age of Abduction	NS	NS	0.98 (0.95, 1.00)	0.035

Abbreviations: OR, odds ratio; CI, confidence interval; PTSD, post-traumatic stress disorder; NA = not applicable because variable was not retained in the final model; UGS, Ugandan Shillings

Odds ratios are adjusted for the effect of other statistically significant variables in the model.

* other group comparison were examined but were also not statistically significant

Multivariate logistic regression analysis showed that meeting symptoms of depression was associated with gender, relationship with family, difficulties returning home, cumulative direct violent traumatic exposure, and cumulative exposure to forced acts of violence (see Table 5). Interactions between age and gender were also significant. Among abductees, women were twice as likely as men to report symptoms of depression (OR_adj _= 2.11, 95% C.I. = 1.22, 3.63). Due to statistical interaction between age and gender, each one-year increase in age is associated with multiplicative increase in odds of having symptoms of depression by 3% among men and decrease in odds of having symptoms of depression by 2% among women. This means that while male abductees may have an increased risk of having symptoms of depression as they age, the risk for female abductees may decrease with age. Higher self-reported positive scores for abductees' relationships with their family, friends, and community were associated with a decrease in odds of meeting the criteria for symptoms of depression (OR_adj _= 0.90, 95% C.I. = 0.83, 0.97). Likewise, reported problems when returning were positively associated with the odds of meeting the criteria for symptoms of depression (OR_adj _= 2.24, 95% C.I. = 1.63, 3.08). The cumulative number of general traumatic exposures (OR_adj _= 1.18, 95% C.I. = 1.03, 1.33) and cumulative number of forced acts of violence (OR_adj _= 2.24, 95% C.I. = 1.63, 3.08) were associated with increased odds of meeting the criteria for symptoms of depression among abductees. The following variables were not statistically associated with either symptoms of PTSD or depression in the multivariate analyses described above: 1) length of abduction, 2) going through a reception center, 3) being married or in partnership relationship.

## Discussion

One-third of the respondents (33%) self-reported having experienced abduction (49% among the Acholi). Our findings further suggest that fewer former LRA abductees passed through the reception centers before returning to their home communities than had previously been reported. This in turn suggests that the number of abducted people in northern Uganda may have been underestimated as the estimates were calculated based on the number of children passing through the reception centers [6,13].

Of those who reported abduction, 426 (45%) were held for less than a day, 199 (21%) were held captive for one to seven days and 122 (13%) were held for between one week and one month. Our findings suggest that young men are specifically selected for long-term conscription: The average age at the time of abduction and the proportion of women decreased with longer periods of abduction. This selection process is highlighted also by how those abducted were returned: Over two thirds (67%) of those held in captivity for less than one day were released by the LRA. The LRA may have abducted as many people as they could during a raid to help them carry stolen goods and also enable them to select the fittest individuals for their army.

The findings suggest LRA soldiers force conscripts to commit violent acts such as killing, beating or looting, possibly as a form of indoctrination ritual [1,5]. These practices may force the recruit to violate their own moral principles and to break from any form of social attachment, ultimately making them more submissive and compliant to the leadership [14]. As a result, civilians have paid a high toll during the conflict, often at the hands of those forcibly recruited.

Based on the results, former LRA abductees confront a range of problems when returning to their communities, including psychological (e.g., "mentally do not feel well") and social (e.g., relationship with family, community) problems. Problems associated with returning home were found to be statistically associated with symptoms of PTSD and depression. However, because this is a cross-sectional study, we cannot determine whether symptoms of PTSD and/or depression led to problems associated with returning home or whether problems associated with returning home elicited symptoms of PTSD and/or depression. The study did not find any relationship between going through the reception center and having symptoms of PTSD and/or depression but found that those who had better relationships with their family, friends, and community were less likely to have symptoms of depression (OR_adj _= 0.90, 95% C.I. = 0.83, 0.97). This supports several qualitative research studies conducted in northern Uganda which found that family and community acceptance and support are vital to psychosocial well-being of former LRA abductees [15]. It shows the value of reintegrating former abductees with their families and developing programs to help them move ahead economically and socially. At the same time, the study did not find a significant association between social relationships and symptoms of PTSD, suggesting that the reintegration process alone is insufficient to address those symptoms. Also the study did not find an association between having attended a reception center and symptoms of PTSD and depression. However, going through a reception centers was found to have helped the reintegration process.

The prevalence of those who met criteria for symptoms of PTSD among former LRA abductees was high: over half of all the respondents and over two-thirds of those who experienced abduction met the criteria for symptoms of PTSD (four out of five among those abducted six months or more). Nevertheless, the overall prevalence of those who met the criteria for symptoms of PTSD among former LRA abductees is lower than suggested in earlier studies [8]. This change may be due to significantly decreased levels of violence since earlier assessments or because people may have learned to cope with their symptoms. The prevalence of symptoms of depression was also high in our sample, with nearly one third of the respondents and 40% of those who experienced abduction meeting the criteria for symptoms of depression (nearly half of those abducted for six months or more).

Symptoms of PTSD were present at a higher rate among individual LRA returnees who had witnessed and been exposed to direct acts of violence. Symptoms of depression were more likely among individuals who had been directly exposed to violence and forced to commit acts of violence. Secondary exposure (e.g. direct exposure of family members) was associated with neither symptoms of PTSD nor symptoms of depression. This suggests that beyond the "dose-effect" model linking increased exposure to violence with an increase in psychological trauma, [16-18] witnessing violence and being forced to commit violence can also lead to increased psychological trauma, as we have suggested in previous studies [8,9]. Finally, the length of abduction was not found to be significantly associated with symptoms of PTSD and depression. However, exposure to violence, including being forced to commit acts of violence increase with length of abduction and were found to be significantly associated with those symptoms.

### Limitations

This study has several limitations. The sampling universe for this study consisted of adults aged 18 or above residing in eight districts in northern Uganda. The districts were selected to represent the ethnic composition of the affected population and exposure to the conflict. Children at the time of the study and abductees who have not returned were not included in the sample. Therefore, the results cannot be generalized to all Ugandans but only represent the adult population from which we sampled. The study further relies on self-reported scales and responses that may have been affected by social desirability and recall errors. To minimize this we did not offer any incentive to participate in the study and used a consent form stressing the confidential and anonymous nature of the interviews. Adapting the language and western concepts used in measuring psychological trauma to non-Western populations may result in trans-cultural errors. However, we consulted with local experts and other researchers during the development of our research instrument, and we had successfully implemented a similar study in northern Uganda in 2005. The purpose of this paper is not to be clinically precise (i.e., coming up with a predictive model), but to obtain a greater understanding of factors that are associated with mental health among those who were formerly abducted by the LRA. We assessed exposure to trauma asking respondents whether they have been exposed to the events listed in Table 2. These events were chosen based on our consultation and prior research. [4,10,12]. These traumatic events do not represent all the possible traumatic events but those that were reported frequently. It may not meet all of the subjective and objective components of Criterion A. Furthermore, we did not assess for functional impairment. We used the PTSD Checklist-Civilian Version (PCL-C), a measure of post-traumatic stress disorder, and 15-item depression section of the Johns Hopkins Depression Symptom Checklist (JHD). The use of standard scales to measure the symptoms of psychological disorders such as PTSD and depression has been widely debated. Critics generally stress the lack of validity of those measurements because they are applied in different cultural environment and in conflict situations [19-21]. Those measures were nevertheless included in our analysis because they are validated [21-23] measures of psychological trauma outside clinical diagnostic interviews and have been applied in war-affected countries, such as the former Yugoslavia, Rwanda, Algeria, Cambodia, and Ethiopia, among other countries [8,9,17,24]. Nonetheless, the instruments were not specifically validated for northern Uganda. In addition, we dichotomized the scores for the criteria for symptoms of PTSD and depression using two cut-off points: symptoms of PTSD (cutoff score of 44) and depression (cutoff score of 42). The two cut-off scores are chosen because they yielded the most conservative estimates and permitted us to conduct logistic regression. It is possible that more respondents had symptoms of PTSD and depression than reported. Finally, causal relationships cannot be established because of the cross-sectional design of this study. Further research will be needed to assess these relationships.

## Conclusion

We found high prevalence rates of symptoms of PTSD and depression among former LRA abductees in northern Uganda. The symptoms were associated with directly experiencing violence, being forced to commit violent acts, and/or witnessing violent acts. All of these symptoms were more frequent among those held captive for longer periods of time. We found that problems returning home were associated with symptoms of PTSD and depression. In addition, those who reported having better relations with their family, friends, and community were less likely to report symptoms of depression.

These findings have important implications for mental health services and reintegration programs in northern Uganda. This study corroborates other studies that found family and community play an important role for the psycho-social well being of former abductees [15]. Thus addressing psychological trauma and helping former abductees reunite with their family and community are paramount to facilitating the return to their communities of those held in captivity.

At the same time, improving the quality of life of former abductees goes beyond psychological care and must include an inclusive livelihood-based approach to improve access to education and economic opportunities. The approach should be aimed at the community as a whole to decrease the level of stigmatization already experienced by some former LRA abductees [13,25,26].

Our study found that few former LRA abductees went through reception centers. Even fewer received follow-up visits by reception center staff that might have helped with their reintegration. Going through a reception center was not statistically associated with a decrease in symptoms of depression or PTSD. This is not surprising because reception centers were not originally established to offer such services. However, should a large influx of former LRA abductees return to northern Uganda, reception centers could provide these returnees with a safe place to stay as they re-adjust to civilian life and are re-integrated with their families and communities.

## Competing interests

The authors declare that they have no competing interests.

## Authors' contributions

PNP and PV designed the study, conducted the field study in Uganda, and analyzed the data. PNP, PV, and ES interpreted the data and wrote the report. All authors read and approved the final manuscript.

## Pre-publication history

The pre-publication history for this paper can be accessed here:


